# Broad-Spectrum Antivirals against Multiple Human and Animal Coronaviruses Infection

**DOI:** 10.3390/pathogens12060823

**Published:** 2023-06-11

**Authors:** Divyasha Saxena, Lalit Batra, Shailendra Kumar Verma

**Affiliations:** 1 Center for Predictive Medicine, University of Louisville, Louisville, KY 40202, USA; divyasha.saxena@louisville.edu; 2 Center for Infectious Disease and Vaccine Research, La Jolla Institute for Immunology, San Diego, CA 92037, USA; skverma@lji.org

Among the seven coronaviruses that infect humans, HCoV-229E, HCoV-OC43, HCoV-NL63, and HCoV-HKU1 usually cause mild and common cold symptoms; however, infection with three coronaviruses, namely severe acute respiratory syndrome coronavirus [SARS-CoV], Middle East respiratory syndrome coronavirus [MERS-CoV], and the newly identified severe acute respiratory syndrome coronavirus 2 [SARS-CoV-2], often results in respiratory distress, cytokine storm and multiorgan failure [1]. Group 2b SARS-like coronaviruses represent a threat to global health, as evidenced by the emergence of SARS-CoV in 2002 and SARS-CoV-2 in 2019 [2]. At present, different plans are being made to effectively contain the virus; these plans are based on the inhibition of viral replication using drugs, neutralizing antibodies or dampening the host response; however, the development of novel anti-coronavirals for all highly pathogenic coronaviruses remains a major challenge. Several in vitro and animal studies have been identified against coronaviruses; still, efficacy data from human clinical trials are desperately needed. 

Milewska et al. have shown the efficacy of chitosan N-(2-hydroxypropyl)-3-trimethylammonium chitosan chloride (HTCC) against multiple human endemic coronaviruses, such as HCoV-NL63, HCoV-229E, HCoV-OC43, and HCoV-HKU1. Pre-treatment of Vero cells with HTCC inhibited MERS-CoV and SARS-CoV-2 viral replication with an IC_50_ (inhibitory concentration) value of 62.8 and 12.5 μg/mL, respectively [3]. Further, pretreatment with HTCC protected human airway epithelial lung tissues (HAE) from MERS and SARS-CoV-2 infection. Animal studies and human clinical trials are required to fully understand the antiviral potential and efficacy of HTCC compounds. 

Timothy et al. evaluated the efficacy of an oral bioavailable compound β-d-N4-hydroxycytidine (NHC; EIDD-1931) against SARS-CoV, SARS-CoV-2, MERS-CoV, and other zoonotic coronaviruses. In this study, the antiviral activity of NHC was evaluated against different bat-CoV using a human airway epithelium model. The researchers observed that pretreatment with NHC prevents SARS-like (HKU3 and SHC014, group 2b) and MERS-like (HKU5, group 2c) bat-CoV infection in HAE tissues. Further, prophylactic and therapeutic treatment with the ribonucleoside analog NHC, EIDD-1931, improved lung pathology and reduced viral titers and clinical scores in SARS-CoV, SARS-CoV-2 and MERS-CoV-infected mice [4]. These data will further support the development of EIDD therapeutics against newly emerged and emerging coronaviruses in the near future.

Using in silico screening and in vitro validation assays, Pendyala et al. deciphered the binding affinity and inhibition of various Phycobilin compounds against towards the inhibition of main protease (M^pro^) and papain-like protease (PL^pro^) of SARS-CoV-2. A sequence analysis of beta coronaviruses revealed that the M^pro^ and PL^pro^ are highly conserved among functional regions; docking analysis further revealed that phycobilin compounds have broad-spectrum coronavirus inhibitor activity, with an IC_50_ values of 71 and 62 μM, respectively. However, in vivo studies using human cells and animal models are required to prove the efficacy of these compounds [5]. 

Griffithsin (GRFT), an alga-derived lectin, has shown broad-spectrum antiviral activity by inhibiting viral entry through the attachment of carbohydrate-binding domains to viral outer surface glycoproteins [6]. O’Keefe et al. have demonstrated the in vitro efficacy of GRFT on multiple human coronaviruses such as OC43, 229E, and NL63 (with EC_50_ values of 52, >10 and 10 μg/mL, respectively), and against different strains of SARS-CoV, viz. Urbani, Tor-II, CuhK, and Frank. Finally, they showed the efficacy of GRFT treatment on mortality and morbidity in a lethal mouse infection model, using mouse-adapted SARS-CoV [7]. The same research group has also reported the efficacy of GRFT treatment against in vitro MERS-CoV infection, using Huh-7, MRC-5 and Vero-81 cells; they reported that a minimal amount (2 μg/mL) of Griffithsin was required for 90% viral inhibition [8]. Recently, Ahan R. E et al. reported the efficacy of Griffithsin against the Delta and Omicron variants of SARS-CoV-2 using IFNAR^–/–^ mouse models, and showed that prophylactic treatment with Griffithsin prevented SARS-CoV-2 infection, blocked viral transmission, and reduced the viral load in mice [9]. 

Nafamostat, a drug used to treat pancreatitis, has been proven efficacious against MERS-CoV [10] and SARS-CoV-2 [11]. Researchers have established that nanomolar concentrations of Nafamostat were most effective iterations of the drug, preventing SARS-CoV-2’s fusion with 293FT cells (human fetal kidney expressing ACE2 and TMPRSS2) and Calu-3 cells (non-small-cell lung cancer). Disulfiram, a drug widely used in treatment of alcoholism, was observed to be a competitive inhibitor of SARS-CoV and an allosteric inhibitor of MERS-CoV’s PLpro protease, thereby blocking their replication [12]. Lopinavir and ritonavir, drugs used in the treatment of HIV, were proven effective against SARS-CoV [13], SARS-CoV-2, and MERS-CoV [14] through various in vitro and animal studies. 

Ribavirin, a drug approved for the treatment of HCV, has been evaluated for patients infected with MERS-CoV and SARS-CoV-2; however, it is often associated with various safety and toxicity concerns [15]. Lopinavir (LPV) and ritonavir (RTV) are FDA-approved HIV-1 protease inhibitors that also appear to inhibit 3CLpro activity. Lopinavir has been shown to inhibit SARS-CoV, MERS-CoV, and SARS-CoV-2, with EC_50_ concentrations of 17.1, 8 and 26 μM, respectively [16]. The MIRACLE trial (MERS-CoV Infection tReated with A Combination of Lopinavir/ritonavir and intErferon-β1b) has evaluated a combination therapy of LPV/RTV + IFN-β compared to placebo in MERS-confirmed hospitalized patients (NCT02845843). Chloroquine and Hydroxychloroquine demonstrated potent in vitro activity against SARS-CoV-2 [EC_50_: 1.13 μM], SARS-CoV-1 [EC_50_: 1–8.8 μm], and MERS-CoV [EC_50_: 3.0 μM] [16]. A double-blinded, randomized, placebo-controlled trial comparing 4652 participants was carried out to evaluate the long-term efficacy (5 months) of Chloroquine/ Hydroxychloroquine treatment (NCT04303507). Emetine, FDA-approved alkaloid used for the treatment of ameobiasis, has shown potent antiviral activities against HCoV-OC43, HCoV-NL43, SARS-CoV-1, SARS-CoV-2, MERS-CoV, and MHV-A59 in vitro at low micromolar concentrations [17].

Remdesivir, another intravenous broad-spectrum drug, has shown antiviral activities against different coronaviruses. GS-5734, a Remdesivir analogue, inhibited MERS-CoV and SARS-CoV replication in human lung epithelial Calu-3 2B4 cells, at sub-micromolar concentrations at least 100-fold lower than cytotoxicity values [18]. Further, prophylactic and therapeutic treatment with GS-5734 reduced virus lung titers, improved lung function (reduced Penh score), and inhibited symptoms of SARS-CoV disease in mice genetically deleted for Ces1c^−/−^ (secreted carboxylesterase 1c) [19]. The antiviral activities of Remdesivir against endemic human CoVs, HCoV-OC43 and HCoV-229E, have been shown at sub-micromolar EC_50_ values [20]. Remdesivir has been shown to inhibit SARS-CoV-2 replication in a rhesus macaque model, with reduced pulmonary infiltrates and virus titers in bronchoalveolar lavages [21]. A phase III randomized open label study of the safety and antiviral activity of Remdesivir was successfully completed on 4891 participants with severe COVID-19 conditions (NCT04292899). Based on these findings, Remdesivir was the first FDA-approved antiviral for the treatment of moderate-to-severe COVID-19 cases, given the green light in October 2020.

In May 2023, the FDA approved the first oral antiviral, Paxlovid, for adult patients at high risk of progression to severe COVID-19, based on the phase II/III EPIC-HR (Evaluation of Protease Inhibition for COVID-19 in High-Risk Patients) study, which enrolled unvaccinated, non-hospitalized adults with confirmed COVID-19 who were at increased risk of progressing to severe disease. The data showed an 86% reduction in the risk of COVID-19-related hospitalization or death by any cause, up to day 28, in patients who initiated treatment with Paxlovid within five days of their symptoms’ onset, as compared to placebo. This drug has been shown to inhibit different SARS-COV-2 variants of concern, such as Alpha, Beta, Delta, Gamma, Lambda, Mu, and Omicron BA.1, BA.2, BA.2.12.1, BA.4, BA.4.6, BA.5, BF.7, BQ.1.11, BQ.1 and XBB.1.5 [22]. 

SARS-CoV-2 mutated several times over the course of its existence, and has evolved into genetically distinct mutants such as Beta, Delta, and Omicron. Most available vaccines are based on the original strain of the virus. Hence, the efficacy of currently developed vaccines against newly emerged and emerging variants is highly doubtful, despite the antibody waning effect post 6 months. Various studies have reported potential inhibitors against SARS-CoV-2; limited data are available on broad-spectrum inhibitors of a wide range of human and animal CoVs. The design and development of broad-spectrum antivirals against coronaviruses is an attractive approach to tackling emerging viral variants. Remdesivir, Molnupiravir, and Ritonavir-boosted Nirmatrelvir (brand-name Paxlovid) are the only antivirals approved for use against COVID-19 disease, with limited data on other coronaviruses available. This editorial welcomes the submission of manuscripts related to broad-spectrum coronavirus antivirals, their mechanism of action, safety, and efficacy.
